# Revisiting Spectrophotometric Methods in the FoodOmics Era: The Influence of Phytochemicals in the Quantification of Soluble Sugars in Plant-Based Beverages, Drinks, and Extracts

**DOI:** 10.3390/foods14162889

**Published:** 2025-08-20

**Authors:** Ana Reis, Cláudia P. Passos, Elsa Brandão, Natércia Teixeira, Tiago Alves, Nuno Mateus, Victor de Freitas

**Affiliations:** 1 REQUIMTE/LAQV, Departamento de Química e Bioquímica, Faculdade de Ciências, Universidade do Porto, Rua do Campo Alegre, 4169-007 Porto, Portugal; 2 REQUIMTE/LAQV, Departamento de Química, Universidade de Aveiro, Campus Universitário de Santiago, 3010-193 Aveiro, Portugal; cpassos@ua.pt

**Keywords:** fermented foods, plant-based milk, bioactive compounds, anthocyanins, melanoidins, carotenoids, chlorophylls, catechins, curcuminoids

## Abstract

The rising prevalence of diet-related diseases is driving consumers to adopt healthier, plant-based diets. Aware of this consumer trend, the Food Industry is investing in innovative, tasty, plant-based foods with added nutraceutical value. However, health-promoting phytochemicals are often found in foods with a high content of natural sugars that are readily absorbed, undermining their health benefits. To ensure proper labelling and support consumers in their choices for healthier foods, the Food Industry relies on cost-effective methods to measure soluble sugars. Herein, we assess three established spectrophotometric assays—phenol, orcinol, and anthrone—for quantifying soluble sugars in 12 plant-based beverages, drinks, and extracts. The standard glucose solutions revealed that the phenol and orcinol reagents displayed the highest sensitivity. Applied to phytochemical-rich beverages, drinks, and extracts, the anthrone protocol leads to precipitation phenomena; the phenol is prone to interference from chlorophylls, carotenoids, melanoidins, (ellagi)tannins, and anthocyanins, whereas orcinol is susceptible only to anthocyanins. Though spectrophotometric assays overestimate sugar levels in both high- and low-sugar-content samples, the orcinol-sulfuric acid method offers an environmentally safe and cost-effective approach to quantifying soluble sugars in phytochemical-rich samples, fostering food innovation and helping to build consumer trust within resilient and sustainable food systems.

## 1. Introduction

In recent years, research has unveiled the benefits that the continuous and sustained intake of plant-based and fermented diets have. They not only lead to improvements in blood lipids and cardiometabolic markers [1,2,3,4], but they also act as key players in microbiota ecology and gut functionality [5,6,7,8], capable of modulating the host immune system [7], resolving low-grade inflammation in age-related diseases [9] with impacts on disease prevention and health promotion. Given the rising incidence of diabetes, particularly in children and young adults [10], consumers are becoming increasingly aware of the impact of their daily food choices on health outcomes and are progressively adopting healthier plant-based diets.

Aware of this plant-based consumer trend, the Food Industry is taking innovative approaches aimed at exploring local seasonal foods and other surplus food for developing tasty food products with nutraceutical properties, contributing to the sustainability of food supply chains. However, the intake of plant-based foods rich in bioactive compounds is often accompanied by the ingestion of natural simple sugars, such as lactose, sucrose, fructose and glucose. These sugars, contrary to other oligosaccharides with beneficial prebiotic effects [11] and complex polysaccharides that contribute to satiety and reduce the absorption of cholesterol in the intestine [12], are rapidly absorbed in the gastro-intestinal (GI) tract, contributing to persistent high sugar levels in the circulation and ultimately undermining the health-promoting potential of plant-based foods.

To protect and support consumers in their healthy food choices, the European Union (EU) has strict labelling legislation [13]. On the other hand, as 99% of food and drinks businesses are SMEs [14], to comply with EU demands, the costs associated with RD&I is restricting food innovation. To remain competitive in the global food market, the Food Industry relies on rapid and cost-effective approaches for the high-throughput quantification of sugars in foods either in the final product or at intermediate steps (e.g., extraction, cooking, evaporation, dilution, mixing, drying, fermentation, distillation) for quality control purposes. Due to their portability, swiftness, and low cost, sugars are routinely quantified by refractometers in the wine, beer, and juice industries. However, as Brix values reflect levels of soluble solids such as sugars and other compounds (e.g., proteins, organic acids, and others) they are often inaccurate, rendering them of little value when applied to foods from new or less explored sources. Alternatively, soluble sugars can be routinely estimated using (enzymatic) commercial kits, advanced analytical techniques (e.g., GC, LC, and NMR) or spectrophotometric methods [15,16,17,18,19]. Both (enzymatic) commercial kits and advanced LC-MS approaches are expensive alternatives, whereas low-cost spectrophotometric approaches require low-grade skilled operators and are thus advantageous.

The quantification of sugars through spectrophotometric approaches typically involves a wet-chemistry step in the presence of concentrated sulfuric acid at high temperatures, resulting in the dehydration of sugar residues to 5-hydroxy-methyl-furfural, which, by reacting with the staining reagent, develop colour, allowing for the quantification of sugars at specific wavelengths. Developed in the 1950s, several staining reagents have been proposed, including anthrone [20], orcinol [21] and phenol [22], and applied to the quantification of sugars in a variety of food matrices such as (micro)algae, seaweed, dragon fruit, rice, and bovine dairy products [17,23,24,25,26]. Despite the diversity of staining reagents and the recognised susceptibility of the phenol-sulfuric acid protocol to interference from chlorophylls and carotenoids [23,26], the phenol reagent remains the most popular protocol. Remarkably, comparative studies on the performance of the different staining reagents in the quantification of sugars in phytochemical-rich matrices are limited.

To address this gap, we evaluate the performance of three chemical-based spectrophotometric assays (phenol, orcinol, and anthrone) in the quantification of soluble sugars in fermented and fresh drinks and beverages containing an array of bioactive health-promoting phytochemicals.

## 2. Materials and Methods

### 2.1. Reagents

D-Glucose (D-Glc, purity > 99.5%), orcinol (97%), anthrone, and phenol (99%) were purchased from Sigma, and sulfuric acid (95–97%) was purchased from Fluka (Madrid, Spain). Ultrapure (MilliQ) water was filtered through a Milli Q Reagent Water System apparatus (InterLab, São Paulo, Brasil), where the specific electrical resistance of water used throughout the experiments was <18.2 MΩ/cm.

### 2.2. Plant and Fruit Samples and Extract Preparation

Fresh and fermented plant-based beverages, drinks, and extracts containing distinct health-promoting phytochemicals were included in this study, namely, chlorophyll-rich aqueous extracts (spinach leaves, mint leaves, and algae), carotenoid-rich (orange juice and spinach leaves), anthocyanin-rich (red wine and elderberry juice), melanoidin-rich (coffee), catechin-rich (Irish tea), and (ellagi)tannin-rich samples (whisky). Beverages with distinct alcohol content (5–40%) were also included to investigate the influence of alcohol on sugar quantification by the various approaches. Details on the origin and experimental preparation undertaken for each sample is described below.

Coffee: Powdered coffee capsule (Buondi, Nestlé) made of medium-roast (intensity 8) Arabica/Robusta coffee beans originating from Africa/Colombia was prepared using an espresso coffee machine (Krupps, Lisboa, Portugal), and poured into 1 small coffee cup (40 mL).

Elderberry juice: Elderberries (*Sambucus nigra*) were harvested (August 2023) and frozen. Approximately 30 g of thawed elderberries was mixed with 50 mL of water and ground using an UltraTurrax (IKA, Staufen, Germany) at 9500 rpm for 60 s at room temperature.

Orange juice: A seedless orange (Baía variety, approx. 380 g) was hand-squeezed and the juice strained.

Algae aqueous extract: Roughly 0.5 g of dry Nori seaweed (Miyata, China) was mixed with 40 mL of water and ground using an UltraTurrax (IKA) at 9500 rpm for 60 s at room temperature.

Mint leaf aqueous extract: Roughly 1.8 g fresh mint leaves (*Mentha spicata*) was mixed with 40 mL of water and ground using an UltraTurrax (IKA) at 9500 rpm for 60 s at room temperature.

Spinach leaf extract: ~5.0 g fresh spinach leaves (*Spinacia oleracea*) was mixed with 50 mL of water and ground using a UltraTurrax (IKA) at 9500 rpm for 60 s at room temperature.

Irish afternoon tea infusion: 1 teaspoon (~3.0 g) of Irish Afternoon tea (Galway, Ireland) was infused in 1 cup (150 mL) of boiling water (>95 °C) for 2 min, after which the tea infusion was left to cool at room temperature.

Ginger/turmeric infusion: Fresh ginger (23.3 g) and turmeric (6.7 g) roots were sliced, infused with 1 L of boiling water, and left for 1 h.

Oat milk: 70 g of rolled oats (Continente, Lisboa, Portugal) was soaked in 1 L of cold water for 45 min, after which they were wet-milled in a high-speed blender.

Whisky: Single malt scotch whisky aged in oak casks for 15 years (Grants, Banffshire, Scotland) containing 40% alcohol.

Beer: Commercial barley malt lager beer (Superbock, Leça do Balio, Portugal) containing 5.2% alcohol was degassed by ultrasound for 30 min.

Red wine: Commercial red wine made from Trincadeira, Aragonez and Castelão varieties (2022) from the region of Alentejo (Reguengos de Monsaraz, Portugal) containing 13.5% alcohol.

When needed, samples (pulp material) were filtered (Whatman GF/A, Ø 125 mm, Cytiva brand, Lisboa, Portugal), centrifuged (13,000 rpm, 10 min) at room temperature and the supernatant collected.

### 2.3. Physico-Chemical Characterisation of Samples

Physico-chemical parameters such as pH, colour, and Brix values were measured. pH values were measured using a WTW 538 pH meter with an Inlab semi-micro sensor (Mettler Toledo, Columbus, OH, USA). CIELab coordinates were determined using a TRA 520 Handheld & Benchtop Sphere Spectrophotometer (Lovibond, UK) with 1 cm glass cells. Spectra in the visible region were recorded using a microplate reader (Bio-Tek, Highland Park, VT, USA). Degree Brix (°Brix) values were obtained using a dual-scale portable optical refractometer 0–25% vol/0–20°, with Automatic Temperature Compensation (Chemillé-en-Anjou, France) and calibrated with distilled water. Using Brix values, degree Baumé (°Bé) values were determined using Equation (1):

(1) $${}^{\circ}Bé=\frac{{}^{\circ}Brix+1.6}{1.905}$$

These values were substituted into Equation (2) to determine Specific Gravity (SG) values:

(2) $$SG=\frac{145}{145-{}^{\circ}Bé}$$

and converted into sugar content (g/L) using Equation (3):

(3) Sugarconcentration = °Brix × SG × 10 × 0.9982,

### 2.4. Quantification of Soluble Sugars by Spectrophotometric Methods

Soluble sugars were estimated by spectrophotometry protocols using 3 staining reagents under acidic conditions, namely, with anthrone [20], orcinol [21], and phenol [22]. The D-glucose (Glc) standard stock solution was freshly prepared (1 mg/mL) in ultrapure water. For the calibration curve, dilution of the Glc stock solution ranging from 0.1 to 1000 ppm was carried out in microtubes. Samples (*n* = 3) of phytochemical-rich matrix were freshly prepared on different days and analysed using spectrophotometric methods. Measurements of freshly prepared samples were carried out on different days in 96-well microplates. For each method, each microplate included the calibration curve (11 concentration points and blank, duplicates) and the 12 samples (duplicates along with one analytical blank). Caution is advised as all 3 protocols involve handling highly concentrated acid solutions incubated at high temperatures (85–100 °C), so protective goggles and gloves must be worn at all times.

Orcinol protocol: Briefly, 30 μL of each calibration solution, sample, or blank (water) was added in a microtube to 270 μL of freshly prepared 0.2% (*w*/*v*) orcinol prepared in H_2_SO_4_ (70%) solution. The mix (total volume 300 μL) was vortexed and incubated for 15 min at 85 °C in a heating block (ThermoScientific, Shanghai, China). After this period, microtubes were left to cool, and the solution was transferred to a 96-well flat-bottomed microplate. For each replicate, absorbance was measured (duplicates) at λ = 505 nm in a microplate reader (Bio-Tek, Highland Park, VT, USA). Absorbance values of each well were plotted against the calibration curve and soluble sugars estimated. The results are expressed as μg sugar/mL solution.

Anthrone protocol: In a microtube, 100 μL of each calibration solution, sample, or blank (water) was added to 500 μL of freshly prepared 0.2% (*w*/*v*) anthrone solution in H_2_SO_4_ (70%). The mix (total volume 600 μL) was vortexed and incubated for 10 min at 95 °C in a heating block (ThermoScientific, Shanghai, China). After this, microtubes were left to cool, and then centrifuged at 5000 rpm for 3 min (MiniSpin, Eppendorf, Hamburg, Germany). The solution was transferred to a 96-well flat-bottomed microplate. Absorbance of replicates (duplicates) was measured at λ = 620 nm in a microplate reader (Bio-Tek, Highland Park, VT, USA). Absorbance values of each well were plotted against the calibration curve and soluble sugars estimated. The results are expressed as μg sugar/mL solution.

Phenol protocol: In a microtube, 50 μL of each calibration solution, sample, or blank (water) was added to 150 μL of H_2_SO_4_ (70%). The mixture was vortexed and 30 μL of phenol reagent (50 mg/mL water) was added. The mix (total volume 230 μL) was vortexed and incubated for 30 min at 98 °C in a heating block (ThermoScientific, Shanghai, China). After this time, microtubes were left to cool to RT for 10 min, and the solution was transferred to a 96-well plate. Absorbance of replicates (duplicates) was measured at λ = 490 nm in a microplate reader (Bio-Tek, Highland Park, VT, USA). Absorbance values of each well were plotted against the calibration curve and the corresponding soluble sugars estimated. The results are expressed as μg sugar/mL solution.

### 2.5. Quantification of Soluble Sugars by Gas Chromatography Coupled with a Flame Ionisation Detector (GC-FID)

Soluble sugars were quantified as alditol acetates by gas chromatography according to the methodology described previously [27]. The methodology was adapted for liquid samples using 20 µL of each sample replicate. The sample was dried under vacuum at room temperature, followed by hydrolysis by the addition of 2 M trifluoracetic acid for 1 h at 120 °C. After, 20 μL of internal standard solution (2-deoxy-glucose, 1 mg/mL) was added. The subsequent steps included a reductive step with sodium borohydride (15% in NH_3_ 3 M) for 1 h at 30 °C, and subsequent acetylation with acetic anhydride (3 mL) in the presence of 1-methylimidazole (0.45 mL) for 30 min at 30 °C to form alditol acetate derivatives. Derivatives were separated from the water phase with several washes with dichloromethane. The alditol acetates were analysed by gas chromatography (GC) with a flame ionisation detector (GC-FID) (Perkin Elmer-Clarus 400) equipped with a 30 m column DB-225 mm (J&W Scientific, Folsom, CA, USA) following the chromatographic conditions described previously [28].

### 2.6. Statistical Analysis

Spectrophotometric data (12 samples, *n* = 3) were analysed by ANOVA using Bonferroni’s test in the software package GraphPad Prism (San Diego, CA, USA, v9.0.0 for MAC OS). Statistical significance was determined at *p*-value thresholds of (^a^) *p* < 0.0005 and (^b^) *p* < 0.0001.

## 3. Results

### 3.1. Performance of Spectrophotometric Methods Using Standard Glucose Solution

**Figure 1:**
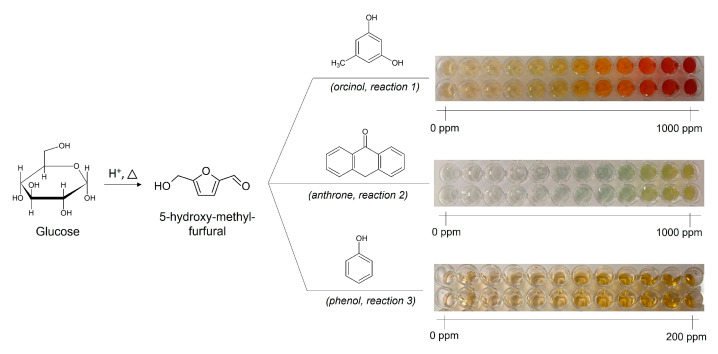
Schematic representation of glucose solution (standard) incubated with orcinol, anthrone, and phenol reagents.

To assess the performance of staining reagents, glucose standard solution (1 mg/mL) was tested with solutions of orcinol and anthrone (0–1000 ppm) and phenol reagents (0–200 ppm), as schematised in Figure 1. The thermal degradation of glucose in the presence of concentrated sulfuric acid results in the formation of 5-hydroxy-methyl-furfural derivatives that, by reacting with the staining reagent, develop a yellowish colour absorbing in the visible light range. The reaction of 5-hydroxy-methyl-furfural with orcinol and phenol generates a yellowish-orange colour, whereas reaction with anthrone generates a greenish colour.

**Figure 2:**
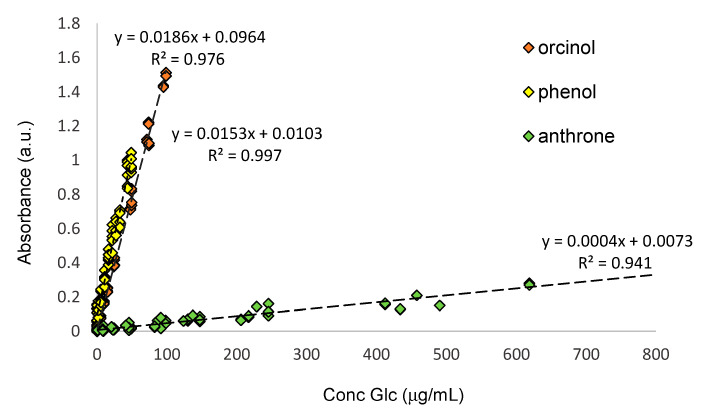
Plot of calibration curve of glucose (standard) obtained for orcinol (orange, λ@ 505 nm), phenol (yellow, λ@ 490 nm), and anthrone agent (green, λ@ 620 nm) protocols.

Because the three protocols involve the use of different volumes of glucose and reagent solution (see Materials and Methods Section), the concentration of glucose (μg of glucose per mL of solution) was normalised for comparison purposes and plotted against the absorbance values, as shown in Figure 2. The results show that phenol-sulfuric acid is the most sensitive protocol (slope = 0.0186), followed closely by the orcinol (slope = 0.0153), with the anthrone protocol as the least sensitive protocol (slope = 0.0004), corroborating the empirical observations (Figure 1).

The higher sensitivity displayed by the phenol-sulfuric acid protocol, compared to the orcinol and anthrone protocols, may be one of the factors contributing to its popularity in the quantification of total sugars [16,24,25,29] and its use as the basis of commercially available kits designed for the quantification of carbohydrates (https://www.sigmaaldrich.com/PT/en/product/sigma/mak104; https://www.abcam.com/products/assay-kits/total-carbohydrate-assay-kit-quantification-ab155891.html; https://www.bioscience.co.uk/product~727693; accessed 20 July 2025). However, phenol is a volatile compound, and its vapours are corrosive to the eyes, skin, and respiratory tract. If in contact with skin, phenol can cause chemical burns. In fact, the median lethal dose (LD_50_) of phenol is 317 mg/kg (rat) and 270 mg/kg (mouse), a lower value when compared to orcinol, which displays an LD_50_ value of 844 mg/kg (rat) [30,31]. Though practical differences exist when handling phenol and orcinol chemicals (Supplementary Information), for the purpose of this study, the three staining reagents were used to measure soluble sugars in plant-based beverages, drinks, and extracts.

### 3.2. Quantification of Soluble Sugars in Plant-Based Beverages, Drinks, and Extracts

To evaluate the applicability of the spectrophotometric methods, a panel of plant-based drinks characterised by distinct bioactive phytochemicals was selected, namely, chlorophyll-rich (mint leaves and algae), anthocyanin-rich (red wine and elderberry juice), carotenoid-rich (orange and spinach leaves), melanoidin-rich (coffee), curcuminoid-rich (ginger and turmeric tea), and catechin-rich samples (Irish tea), as well as grain-based alcoholic drinks (beer and whisky).

#### 3.2.1. Physico-Chemical Characteristics of Plant-Based Beverages, Drinks, and Aqueous Extracts

The physico-chemical parameters, including pH, colour (CIELab coordinates), and Brix values, were measured and are summarised in Table 1. As shown in Table 1, the pH values of the beverages, drinks, and extracts range between 3.5, in red wine, and 7.5, in ginger/turmeric tea infusion.

Similarly, the CIELab coordinates (L*, a* and b*) show a wide variability of colours including brown, grey, red, and violet tones. The lightness coordinate L*, which ranges from 0 (black) to 100 (white), shows that the darker samples are indeed coffee, red wine, and elderberry juice, and the lighter samples are orange juice, ginger/turmeric infusion, and oat milk. On the other hand, the a* and b* coordinates represent the colour’s position along the red-green and yellow-blue axis, respectively, where the most reddish-brown sample is Irish afternoon tea (fermented leaves), with the high positive a* and b* values corresponding to the contributions of red and yellow, respectively. The contribution of green in the algae, mint, and spinach leaf extracts is nearly inexistent due to the removal of intact chloroplast organelles during the sample filtration step. The sugar content was estimated by the measurement of the Brix value using a portable optical refractometer device. Alcoholic beverages (e.g., whisky, red wine, and beer) together with coffee and orange juice showed the highest values, while aqueous samples such as oat milk, coffee, algae extracts, and mint and spinach leaf extracts displayed the lowest Brix values (<1°), thus being indicative of lower sugar content.

#### 3.2.2. Evaluation of Spectrophotometric Protocols in the Quantification of Soluble Sugars

During the quantification of soluble sugars, sample precipitation occurred during the heating step (95 °C, 10 min) in the anthrone protocol. Due to the high variability in the absorbance values of samples and the lower sensitivity of the anthrone protocol (Figure 2), this was not further pursued. For the orcinol and phenol protocols, the dilution factor applied to each sample varied between 5- (for mint and spinach leaves) and 1000-fold (for orange juice). Due to the variable dilution factor applied, some samples displayed colour. To exclude the contribution of coloured non-sugar compounds to the overall absorbance of the extracts at set wavelengths (see Materials and Methods Section), additional blanks containing the diluted sample and H_2_SO_4_ solution in the absence of staining reagent (sample + H_2_SO_4_) were also employed to exclude matrix interference. These procedural blanks (herein named A*_0_) differ from the typical analytical blanks (A_0_), where sample is replaced by water (water + H_2_SO_4_ + staining agent). The absorbance values of the analytical blanks (A_0_) and of the procedural blanks (A*_0_) for the orcinol and phenol assays are summarised in Supplementary Information Table S1.

As can be seen, the analytical blanks (A_0_) in both the orcinol and phenol-sulfuric acid protocols show low absorbance values, whereas the procedural blanks (A*_0_) display strong absorbance for specific samples. In the case of the orcinol protocol, only red wine and elderberry juice display high A*_0_ absorbance when compared to the analytical blank (A_0_), contributing to the overall absorbance at λ = 505 nm (Supplementary Information, Table S1). Both red wine and elderberry juice are known anthocyanin-rich food matrices [32,33] and thus the absorbance value in the absence of staining reagent is likely due to the formation of flavylium ions under acidic conditions with maximum absorbance at λ = 520 nm [34]. For the phenol-sulfuric acid assay, a broader number of samples display high absorbance values for the procedural blanks (A*_0_), namely, coffee, mint, algae, spinach, Irish tea, red wine, whisky, and elderberry samples (Supplementary Information, Table S1), proving the phytochemicals contained in them to be interfering substances in the phenol-sulfuric acid assay, namely, chlorophylls, carotenoids, melanoidins, catechins, anthocyanins, and (ellagi)tannins. Although others have previously reported the potential interference of organic acids, chlorophylls, lipids [23], and carotenoids [26], our study highlights that other phytochemicals, including anthocyanins, melanoidins, catechins, and (ellagi)tannins, are equally likely to affect sugar quantification when using the phenol-sulfuric acid protocol. The development of colour in the absence of a staining reagent requires an additional procedural blank, increasing the time and costs associated with the analysis. When using the phenol staining reagent, the development of colour may be due to secondary oxidation/condensation reactions involving phytochemicals during the heating step in strong acidic conditions. Complementary spectrophotometric A*_0_ readings carried out with epigallocatechin-gallate (EGCG) and ellagic acid standard solutions suggest the high A*_0_ value observed for the Irish tea and whisky samples (Supplementary Information, Table S1) could be attributed to the epimerization of catechins with oxidation/condensation [35,36] and the degradation of migrating C-(ellagi)tannins from oak barrels during ageing [37], promoted by the heating step under acidic conditions [38]. Curiously, when using the orcinol protocol, colour was not observed for the EGCG and ellagic acid standard solutions. Hence, to minimise the overestimation of soluble sugars from interfering phytochemicals in the different samples, where A*_0_ >> A_0_, the A*_0_ value was subtracted from the samples’ absorbance values (Supplemental Table S1). The estimates of soluble sugars using the phenol and orcinol procedures against the calibration curves are summarised in Table 2. With few exceptions, the coefficient of variation (CV) of the replicates was generally <10%, with the mint and whisky samples requiring the lowest dilution factor (Supplemental Table S1), displaying the highest CV (9.3 and 9.4%, respectively).

The data from Table 2 show good agreement for samples with low sugar content (e.g., ginger/turmeric tea, algae, spinach, and mint leaf extracts), corroborating the conclusion drawn earlier (Figure 2). On the other hand, the estimated soluble sugars for coffee, red wine, beer, and elderberry and orange juices (Table 2) show values with statistical differences. As the soluble sugars in each sample are likely due to the presence of different oligosaccharides and simple sugars, the differences noted in the amount estimated could be due to the response factor of each mono-, di-, and oligosaccharide present in the samples [39].

#### 3.2.3. Quantification of Sugars by Gas Chromatography with Flame Ionisation Detector (GC-FID)

Given the discrepancies in the sugar content between the spectrophotometer protocols for the plant-based beverages, drinks, and extracts with high sugar contents (Table 2), GC-FID analyses were carried out on all of the samples. GC-FID analysis has been extensively employed in the determination of sugar content in food samples [25,28,40,41], and provides information not just on the total sugar content but also on the sugar residue composition. The results obtained are summarised in Supplementary Table S2.

The natural sugars present in fruits and juices are typically simple sugars, such as lactose, sucrose, fructose, and glucose, conferring sweetness. Fructose cannot be directly measured with the chromatographic method applied [40] and is estimated as the sum of mannitol and glucitol, using its epimerization ratio during the reduction step for sugar content quantification. The results obtained from GC-FID show that for most of the samples, Glucose is the main sugar residue in their compositions, except for coffee, which has a monomeric composition rich in galactose (Gal, 43 mol%), mannose (Man, 27 mol%), and arabinose (Ara, 21 mol%), consistent with the presence of galactomannans and arabinogalactans [41], and red algae “Nori” extract, which is rich in Gal (97 mol%), consistent with the presence of sulphated galactans [42]. Similarly, oat milk showing the presence of Glc (22 mol%) and fructose (27 mol%) is consistent with the presence of β-glucans, frutans (fructo-oligosaccharides), maltose (starch degradation), and sucrose, as previously reported in oat milk alternatives [43,44]. The presence of Gal (23 mol%) and Ara (19 mol%) suggests the contribution of oligosaccharides not yet reported in unsweetened oat milk [43,44].

The total sugar content (μg/mg solids) present in each sample weighed (mg/mL), as shown in Supplemental Table S2, enabled an estimation of the total amount of sugars (mg/mL), as summarised in Table 2. For evaluation purposes, Table 2 also includes the values obtained by spectrophotometric methods, as well those obtained with the refractometer. The GC-FID data reveal that beer and elderberry and orange juice exhibit the highest sugar content and are in good agreement with the findings from rapid and cheap spectrophotometry-based assays. The most discordant findings were obtained when using the industrially popular refractometer, showing that whisky followed by red wine, beer, and elderberry and orange juice were the samples with the highest sugar contents, evidencing the limitations of the refractometer in beverages with high alcohol contents (e.g., whisky (40%, *v*/*v*) and red wine (13.5% alcohol)). Sugars in whisky and red wine result from the processing of grains and grapes that are further fermented by yeast to produce the alcohol [45], though in the case of whisky, most of the sugars are removed during the distillation process, leaving only residual amounts, as found through spectrophotometry and GC-FID procedures (Table 2).

## 4. Discussion

Aligned with the consumer’s desire for natural “clean label” products, plant-based foods are accessible alternatives that promote health and wellbeing. Individually, the samples selected are enriched in distinct phytochemicals that, due to their health-promoting properties, are often included in herbal infusions and functional beverages (e.g., ginger, turmeric, spinach, orange, mint, algae, and elderberry) and as alternatives to animal-based milk (oat).

The results from our study show that phytochemicals have a major influence on the quantification of soluble sugars when using the phenol-sulfuric acid protocol. The higher susceptibility of the phenol reagent to interference from phytochemicals (Supplemental Table S1) has, with few exceptions [26], been generally overlooked in the literature. The higher susceptibility of the phenol reagent together with the health and environmental hazards associated with the use of phenol renders orcinol as a more environmentally friendly, safer, and more swift colorimetric protocol for the quantification of soluble sugars when screening phytochemical-rich samples.

Even though the experimental apparatus in this lab-based study involved the preparation of the reaction mix in microtubes, which were then transferred to 96-well microplates for stirring, heating, and absorbance readings, given the simplicity and low interference from most phytochemicals, the orcinol spectrophotometric-based assay shows potential for automation for the scaled-up screening of phytochemical-rich sample batches in industrial scenarios. This can be facilitated by the automated pipetting of samples and orcinol reaction solution into heat-resistant polypropylene 384-well microplates at up to 150 °C, which, after stirring, can be placed in heating blocks for subsequent absorbance reading. Preparation as one continuous step opens the opportunity for a cost-effective option to flag phytochemical-rich samples with low soluble sugar content. These samples can then be further characterised by advanced analytical techniques in the development of functional drinks tailored for the (pre-)diabetic population. This has particular relevance considering the increasing efforts in the exploratory valorisation of endogenous native fruits and (medicinal) plants from low-income countries with limited access to costly and highly skilled LC-HILIC-MRM-MS strategies [46] and GC techniques [29].

## 5. Conclusions

Spectrophotometric assays employing a reaction with the staining reagents phenol, orcinol, and anthrone were evaluated in the quantification of soluble sugars in plant-based drinks and beverages containing a range of health-promoting phytochemicals.

The results from this study indicate that both the phenol and orcinol reagents displayed high sensitivity, with the phenol reagent exhibiting a higher susceptibility to interference from phytochemicals (anthocyanins, chlorophylls, carotenoids, melanoidins, catechins, and (ellagi)tannins). Due to phenol’s known safety and environmental concerns, orcinol is a simple and environmentally safer alternative for the quantification of soluble sugars in alcoholic and non-alcoholic plant-based beverages, drinks, and extracts. Based on the findings from this study, the orcinol-sulfuric acid spectrophotometric approach can be implemented in industry with clear advantages over the portability and the poor performance of the refractometer, providing a more accurate reading in a more cost-effective manner when compared to expensive commercial kits and advanced chromatographic strategies. This, along with orcinol’s automation potential, suggests the orcinol protocol as a time- and cost-effective alternative for the high-throughput quantification of soluble sugars in the development of innovative functional plant-based food products tailored towards the (pre)diabetic population.

## Figures and Tables

**Table 1:** Physico-chemical characteristics of fermented and fresh plant-based beverages, juices, and extracts. Values relate to mean ± standard deviation (*n* = 3).

	Samples	pH	CIELab Colour	CIELab Parameters			°Brix	Sugar *^#^* (g/L)
				L*	a*	b*		
*Fermented drinks*	Coffee (expresso)	5.30 ± 0.01		24.35 ± 0.02	1.59 ± 0.01	1.11 ± 0.04	2.6	26.4
	Red wine	3.49 ± 0.01		24.47 ± 0.01	4.36 ± 0.03	1.60 ± 0.01	4.6	47.0
	Beer	4.05 ± 0.01		54.78 ± 0.01	2.03 ± 0.02	24.74 ± 0.02	3.8	38.7
	Whisky	3.96 ± 0.01		52.11 ± 0.01	4.99 ± 0.01	32.5 ± 0.04	7.8	80.6
	Irish afternoon tea	4.77 ± 0.02		42.75 ± 0.08	16.83 ± 0.01	33.62 ± 0.02	0.8	8.1
*Fresh drinks*	Ginger tea	7.49 ± 0.04		57.89 ± 0.01	−2.36 ± 0.01	25.54 ± 0.01	0.2	2.0
	Elderberry juice	4.06 ± 0.01		24.01 ± 0.03	1.85 ± 0.00	0.75 ± 0.00	5.2	53.2
	Orange juice	3.92 ± 0.02		61.58 ± 0.02	−1.50 ± 0.00	8.63 ± 0.03	6.4	65.8
	Oat milk	7.09 ± 0.01		56.98 ± 0.01	−0.05 ± 0.01	10.51 ± 0.01	0.6	6.0
	Algae extract	6.23 ± 0.02		48.49 ± 0.01	6.26 ± 0.01	26.23 ± 0.03	0.6	6.0
	mint leaves extract	6.76 ± 0.01		37.74 ±0.01	5.44 ± 0.02	17.59 ± 0.03	0.5	5.0
	Spinach leaves extract	5.96 ± 0.01		48.89 ± 0.01	2.68 ± 0.01	23.56 ± 0.03	0.8	8.1

^#^ sugar values were estimated using Equation (3).

**Table 2:** Summary of soluble sugar content estimated in the plant-based drinks, beverages, and extracts by refractometer, spectrophotometric protocols, and chromatographic (GC-FID) protocol. Values relate to mean ± standard deviation (*n* = 3).

		Sugar Quantification (mg/mL)			
	Samples	Refractometer *	Spectrophotometric Assays		Chromatography Method
			Phenol	Orcinol	
*Fermented* *Drinks*	Coffee (espresso)	26.4	0.30 ± 0.03 ^a^	0.63 ± 0.02 ^a^	0.205 ± 0.003
	Red wine	47.0	0.82 ± 0.07 ^b^	0.42 ± 0.04 ^b^	0.217 ± 0.002
	Beer	38.7	8.27 ± 0.35 ^b^	2.79 ± 0.17 ^b^	2.26 ± 0.09
	Whisky	80.6	0.121 ± 0.004	0.034 ± 0.003	0.034 ± 0.004
	Irish tea	8.1	- ^(♦)^	0.118 ± 0.002	0.06 ± 0.02
*Fresh drinks*	Ginger tea	2.0	0.072 ± 0.008	0.062 ± 0.005	0.029 ± 0.07
	Elderberry juice	53.2	18.7 ± 1.37 ^b^	4.57 ± 0.28 ^b^	1.88 ± 0.02
	Orange juice	65.8	35.2 ± 1.74 ^b^	8.12 ± 0.38 ^b^	6.85 ± 0.19
	Oat milk	6.0	0.19 ± 0.02	0.25 ± 0.01	0.062 ± 0.003
	Algae ext.	6.0	0.23 ± 0.02	0.24 ± 0.02	0.142 ± 0.004
	Mint Leaves ext.	5.0	0.018 ± 0.005	0.022 ± 0.002	0.009 ± 0.003
	Spinach leaves ext.	8.1	0.019 ± 0.002	0.039 ± 0.003	0.004 ± 0.001

* Calculated using the data reported in Table 1. ^(♦)^ value could not be calculated due to A*_0_ >> A_0_. ext.—extract. (^a^) *p* < 0.0005; (^b^) *p* < 0.0001.

## Data Availability

The authors declare that the data supporting the findings of this study are available within the paper and its Supplementary Information files. Any raw data are available upon request from the corresponding author.

## Supplementary Materials

The following supporting information can be downloaded at https://www.mdpi.com/article/10.3390/foods14162889/s1: Supplementary Information: Photos depicting weighing spatula with orcinol crystals (A) and phenol crystals (B). The chemical safety symbols for orcinol (harmful) and phenol (toxic, corrosive, health hazard, environmentally damaging) are also shown (from top to bottom); Table S1: Absorption measurements of analytical blanks (A_0_) for orcinol and phenol protocols, and procedural blanks (A*_0_) of analysed samples for orcinol and phenol protocols. Values depict mean ± standard deviation (S.D., *n* = 3). Cells in grey represent samples where A*_0_ >> A_0_; Table S2: Solids (mg/mL) and sugar content (μg/mg solids) composition estimated in the plant-based beverages, drinks, and extracts. Values relate to mean ± standard deviation (*n* = 3). Sugar residues: Rha—rhamnose, Ara—arabinose, Xyl—Xylose, Fru—Fructose, Man—mannose, Gal—galactose, Glc—Glucose.
