# Supplementary material for: Revisiting Spectrophotometric Methods in the FoodOmics Era: The Influence of Phytochemicals in the Quantification of Soluble Sugars in Plant-Based Beverages, Drinks, and Extracts
Source: Foods. 2025 Aug 20;14(16):2889. doi: 10.3390/foods14162889 (PMC12385314; doi:10.3390/foods14162889)
Supplement: Supplementary file 1 [file foods-14-02889-s001.zip › foods-3811913_Supplemental Information.pdf]

Supplementary Information

Revisiting spectrophotometric methods in the FoodOmics era: influence of phytochemicals in the quantification of soluble sugars in plant-based beverages, drinks and extracts

Ana Reis[1]*, Claúdia P. Passos[2], Natércia Teixeira[1], Elsa Brandão[1], Tiago Alves[1], Nuno Mateus[1], Victor de Freitas[1]

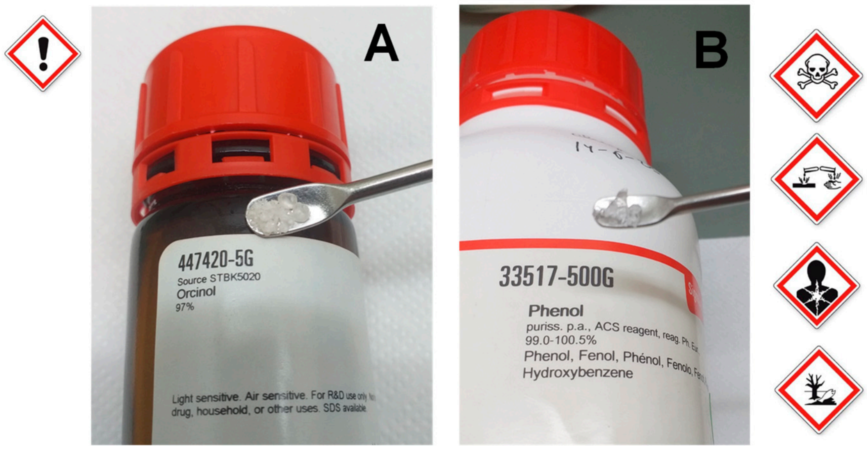

Photos depicting weighing spatula with orcinol crystals (A) and phenol crystals (B). The chemical safety symbols for orcinol (harmful) and phenol (toxic, corrosive, health hazard, environmentally damaging) are also shown (from top to bottom).

From a user's perspective, phenol is a hygroscopic colourless-to-white solid that adheres to the weighing spatula, increasing the time of weighing and the risk of exposure to its harmful vapours, unlike orcinol that is a yellow non-sticky powder that is easily and rapidly weighed.
