# Supplementary material for: Revisiting Spectrophotometric Methods in the FoodOmics Era: The Influence of Phytochemicals in the Quantification of Soluble Sugars in Plant-Based Beverages, Drinks, and Extracts
Source: Foods. 2025 Aug 20;14(16):2889. doi: 10.3390/foods14162889 (PMC12385314; doi:10.3390/foods14162889)
Supplement: Supplementary file 1 [file foods-14-02889-s001.zip › foods-3811913_Supplemental Table S1.pdf]

Supplementary Table S1. Absorption measurements of analytical blanks (A_0) for orcinol and phenol protocols, and procedural blanks (A_0^*) of analysed samples for orcinol and phenol protocols. Values depict mean±standard deviation (S.D., *n*=3). Cells in grey represent samples where $A^*_0 >> A_0$.

	**Blank (A_0)**	**Blank (A^*_0)**											
		**coffee**	**Oat milk**	**mint**	**alga**	**spinach**	**Irish tea**	**Ginger turmeric tea**	**Red wine**	**Whisky**	**Beer**	**Orange**	**Elderberry juice**
phytochemicals		melanoidins	avenanthramides	chlorophyll	chlorophyll	carotenoid	catechins	curcuminoids	anthocyanins	ellagitannins	phenolics	carotenoid	anthocyanins
dilution factor		50	100	5	20	10	20	20	50	5	400	1000	800
**Orcinol (λ=505nm)**	0.082±0.002	0.068±0.004	0.051±0.001	0.058±0.002	0.064±0.003	0.056±0.001	0.072±0.001	0.051±0.001	0.214±0.001	0.071±0.001	0.061±0.002	0.069±0.001	0.116±0.006
**Phenol (λ=490 nm)**	0.075±0.020	0.164±0.001	0.059±0.004	0.127±0.002	0.134±0.001	0.119±0.001	0.391±0.003	0.074±0.002	0.247±0.006	0.272±0.005	0.068±0.002	0.074±0.005	0.118±0.001

A_0: $H_2O+H_2SO_4$+staining agent

A_0^*: sample+H_2SO_4-staining agent
