# Supplementary material for: Revisiting Spectrophotometric Methods in the FoodOmics Era: The Influence of Phytochemicals in the Quantification of Soluble Sugars in Plant-Based Beverages, Drinks, and Extracts
Source: Foods. 2025 Aug 20;14(16):2889. doi: 10.3390/foods14162889 (PMC12385314; doi:10.3390/foods14162889)
Supplement: Supplementary file 1 [file foods-14-02889-s001.zip › foods-3811913_Supplemental Table S2.pdf]

Supplemental Table S2. Solids (mg/mL) and sugar content (μg/mg$_{solids}$) composition estimated in the plant-based beverages, drinks and extracts. Values relate to mean ± standard deviation (*n*=3). Sugar residues: Rha – rhamnose, Ara - arabinose, Xyl – Xylose, Fru – Fructose, Man - mannose, Gal - galactose, Glc – Glucose.

	**Samples**	**Solids content (mg/mL)**	**Sugar composition (%, mol)**							**Sugar content (μg/mg solids)**
			Rha	Ara	Xyl	Fru*	Man	Gal	Glc	
Fermented Drinks	Coffee (espresso)	14.7	tr.	20.9±2.2	-	-	26.8±1.0	**42.9**±1.9	7.5±3.0	13.96±0.02
	Red wine	36.5	-	20.8±0.2	4.5±0.2	13.7±0.2	-	16.0±1.5	**45.0±2.0**	5.95±0.05
	Beer	21.0	tr.	2.0±0.3	2.3±0.2	-	0.8±0.1	0.4±0.1	**94.4**±0.4	100.0±4.2
	Whisky	11.9	-	10.7±1.8	4.8±3.1	-	11.8±2.0	tr.	**72.8**±2.7	2.89±0.24
	Irish tea	3.6	tr.	24.9±3.6	tr.	-	8.6±1.5	10.0±1.5	**54.6**±2.7	15.85±4.2
Fresh drinks	Ginger tea	0.4	tr.	3.0±1.7	1.8±0.5	-	8.3±1.2	3.8±1.3	**83.0**±0.4	72.3±17.6
	Elderberry juice	62.0	tr.	tr.	tr.	6.3±0.3	-	0.8±0.1	**89.9**±4.1	30.4±0.06
	Orange juice	90.8	-	tr.	-	16.9±3.1	-	-	**83.0**±3.1	75.4±2.0
	Oat milk	3.8	-	19.4±1.2	8.5±0.9	**27.0**±4.2	-	23.5±1.2	21.7±5.7	16.4±0.77
	Algae ext.	1.8	-	-	-	-	tr.	**99**±0.8	tr.	78.8±2.0
	Mint Leaves ext.	0.7	-	-	-	-	16.4±9.7	23.1±1.5	**60.5**±8.2	13.1±3.8
	Spinach leaves ext.	2.4	-	-	-	-	19.5±8.6	**40.4**±3.1	40.1±5.2	1.8±1.2

Ext. – extract; n.d. – not determined; tr. - traces.

*Fru was estimated as the sum of mannitol and glucitol using its epimerization ratio during the reduction step.
